# The revival of vaginal surgery in the era of endoscopy: V-NOTES initial experience with a series of 32 patients

**DOI:** 10.52054/FVVO.15.1.064

**Published:** 2023-03-31

**Authors:** J Raquet, L Namèche, M Nisolle, F Closon

**Affiliations:** Department of Obstetrics and Gynaecology, University of Liège, CHR Liège, 4000 Liège, Belgium

**Keywords:** Natural orifice transluminal endoscopic surgery (NOTES), transvaginal natural orifice transluminal endoscopic surgery (V-NOTES), minimally invasive surgery, gynaecologic surgery, hysterectomy

## Abstract

**Background:**

Transvaginal natural orifice transluminal endoscopic surgery (V-NOTES) is an emerging surgical technique in the evolution of minimally invasive surgery. This technique allows different types of surgical procedures to be performed by vaginal access with endoscopic control. The combination of vaginal surgery and laparoscopy brings many advantages, especially the absence of incisions in the abdominal wall and better visualization of the abdominal cavity.

**Objectives:**

In this retrospective study we report our initial experience of V-NOTES in benign gynaecologic surgery by describing our first consecutive 32 surgeries.

**Materials and Methods:**

From June 2020 to January 2022, 32 gynaecological procedures were performed by V-NOTES by the same surgeon in a university hospital. Perioperative outcomes were evaluated retrospectively.

**Main Outcome Measures:**

Conversion to laparoscopy or laparotomy and peri-operative and post-operative complications.

**Results:**

None of the 32 V-NOTES procedures required conversion to conventional laparoscopy or laparotomy. We observed 2 intraoperative complications managed by V-NOTES and 2 post-operative complications (Clavien-Dindo Grade 2).

**Conclusion:**

Our results are similar to studies previously published about this subject and are promising concerning the techniques efficacy and safety. We do believe that a short training allows to reach benefits safely. However, further prospective multicentre randomized trials comparing V-NOTES to totally laparoscopic hysterectomy and to vaginal hysterectomy are needed to strengthen the validity of this new approach.

**What's new?:**

V-NOTES widens the indications of vaginal hysterectomies by removing limitations such as large uterus, absence of prolapse, and history of caesarean. Moreover, this technique allows adnexal surgery to be performed by vaginal access.

## Introduction

Gynaecological surgery is constantly evolving to enhance quality of care and improve patient safety and satisfaction. From the patient’s point of view, the goal is to have a safe and painless procedure with a good aesthetic result. For the surgeon, the goal is to perform a safe, easy, fast and, if possible, ergonomic procedure. Finally, from an economic point of view, the aim is to limit the cost of hospital stays, by reducing the duration of hospitalisation, the costs linked to the intervention and the costs linked to work incapacity.

Hysterectomy for benign disease is one of the most frequent gynaecological procedures. In the USA, 600.000 hysterectomies are performed per year and 80.000 in France. Several surgical approaches are possible to perform this surgery. The first hysterectomy reported was performed by a vaginal access in the ancient times by Soranus of Ephesus for amputation of severely prolapsed uteri. But the first planned hysterectomy was performed by Osiander in Germany in 1801 by a vaginal approach (Senn, 1895). Fifty years later, with the development of the anaesthesia, hysterectomies were performed by laparotomy. Then emerged the minimally invasive surgery (MIS) in the 1940s with the introduction of laparoscopic surgery. The first laparoscopic hysterectomy was successfully performed in 1989 by Reich and colleagues.

The four current approaches for hysterectomy are abdominal hysterectomy (AH), vaginal hysterectomy (VH), laparoscopic hysterectomy (LH) either totally laparoscopic (TLH) or laparoscopy- assisted (LAVH) and robotic-assisted hysterectomy (RH). A Cochrane review by Aerts et al. (2015) recommends VH as the preferred technique when technically feasible, as it is the minimal-access procedure of choice. Vaginal hysterectomy involves shorter hospitalisation time, lower cost, and faster recovery.

The development of MIS has allowed a considerable change in surgical practice over the last 30 years in order to manage patients less aggressively. MIS has introduced new ways to enter the cavity to reduce morbidity, postoperative pain and length of hospital stay while providing aesthetic benefit. Minimally invasive instruments, image guided surgery, robotic surgery, and sutures first emerged in the 1980s and 1990s. Then, in the early 2000s, laparoscopic single incision surgery (SILS, single incision laparoscopic surgery) and transluminal endoscopic surgery using natural orifices (NOTES, transluminal orifice endoscopic surgery) have arisen. In NOTES, the natural orifices of the human body (mouth, rectum, urethra and/or vagina) are used to access the abdominal cavity to perform surgery, thereby avoiding abdominal wall incisions.

NOTES was firstly described by Kalloo et al. (2004). They evaluated trans gastric peritoneoscopy in a porcine model. Then, the first trans gastric appendectomy was performed in humans, which aroused worldwide interest in NOTES technique (Reddy and Rao, 2004).

Transvaginal route quickly surpasses other transluminal access routes. Indeed, transvaginal appears to be the most convenient and safe transluminal route, in comparison with the trans gastric and trans anal routes. The safety and efficacy of colpotomy, the easier closure of the colpotomy incision with little concern for postoperative infection and the use of colpotomy to extract large specimens are advantages of the transvaginal route. The vaginal route is therefore the first to be adopted in clinical practice (Santos and Hungness, 2011).

V-NOTES hysterectomy is performed by a vaginal access using a vaginal port which allows a pneumoperitoneum. Trocars are placed through the port and in these are introduced the camera and the instruments to perform the surgery under visual control. In this way the hysterectomy is more precise and faster. Moreover, this technique allows adnexal surgery to be performed simultaneously.

Su et al. (2012) published the first series of women undergoing transvaginal V-NOTES hysterectomy in 2012. Then, many authors have issued their experiences of V-NOTES in a series of gynaecologic procedures. The main results showed that V-NOTES was a feasible and safe surgical technique for performing adnexal surgeries and hysterectomies even for large and non-prolapsed uterus. This technique combining endoscopy with a vaginal access offers several advantages such as a short operating time, short hospital stays, minimal pain, and no abdominal scar. V-NOTES at least offers similar surgical outcomes and superior cosmesis compared with laparoscopy. (Ahn et al., 2012; Yang et al., 2014; Baekelandt, 2015; Wang et al., 2015)

The first randomized controlled trial comparing V-NOTES hysterectomy with TLH for benign diseases was reported by Baekelandt and Kupurubandara (2019). 35 hysterectomies were performed by V-NOTES and 35 by laparoscopy, none of them required conversion either by laparoscopy for the V-NOTES group or by laparotomy for both groups. They demonstrated that V-NOTES was non-inferior to TLH for successfully performing hysterectomy. Moreover, the hospital stay was shorter after V-NOTES surgery compared to TLH (0.8 vs 1.3 days) and more women left the hospital within 12 hours after V-NOTES surgery compared to TLH (77% vs 43%). Therefore, this technique may also allow more women to be treated in a day-case setting.

In this manuscript, we present our preliminary experience about the V-NOTES technique for benign gynaecological procedures. The aim of this study is to evaluate the efficacy and the safety of this technique. And according to our results, adapt our future practice.

## Methods

From June 2020 to January 2022, we performed 32 gynaecological surgeries using the V-NOTES in a university hospital. This series represent our initial and consecutive V-NOTES procedures.

All procedures were performed by the same gynaecological surgeon experienced in vaginal and laparoscopic surgery and trained specifically for the V-NOTES procedure.

The surgical technique for hysterectomy can be performed using 2 techniques: either vaginally assisted NOTES hysterectomy (VANH) or total vaginal NOTES hysterectomy (TVNH). In this study, all the procedures were performed by the VANH technique.

The first step is the vaginal phase. The table is in a 0° position. Two vaginal retractors are placed anteriorly and posteriorly to the uterus. The opening of the anterior and posterior fornix is performed as in vaginal surgery. It consists in making circumcision of the vaginal mucosa around the cervix and performing an anterior and posterior colpotomy. The uterosacral ligaments are grasped with Jean Louis Faure forceps, cut, and tied off with a Polysorb 1 suture. The suture and needle are kept in a Mosquito clamp to the side of the operating field. The first step allows the access to the peritoneal cavity.

**Figure 1:**
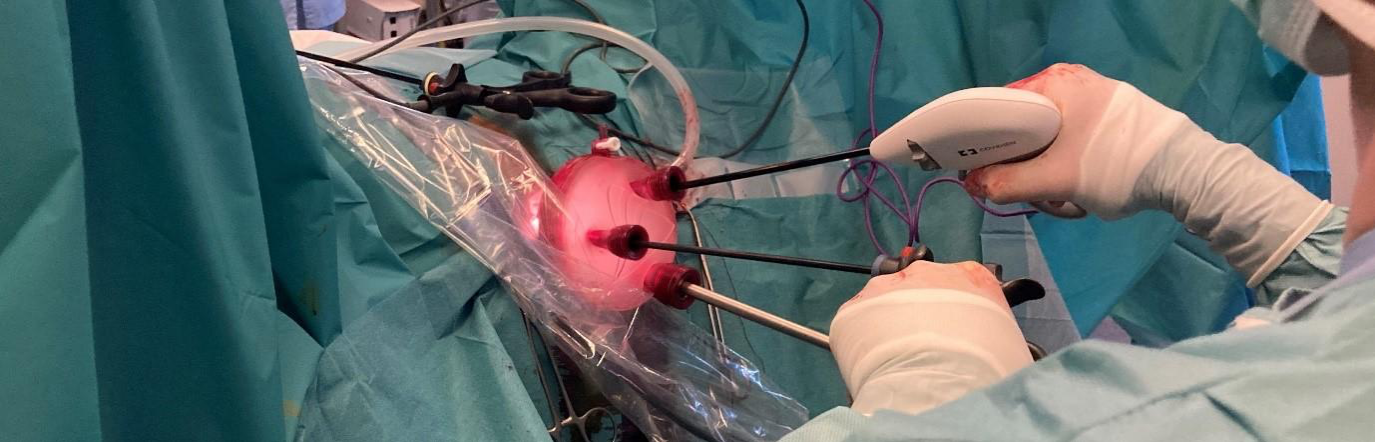
Surgeon performing a hysterectomy with the V-NOTES port.Surgeon performing a hysterectomy with the V-NOTES port.

The second step consists of creating an operating path through the vagina to introduce the V-NOTES port. First the Alexis is inserted through the vagina into the peritoneal cavity. Then the GelPOINT V-Path® is placed over the Alexis to maintain a pneumoperitoneum with an operating pressure of 10mmHg utilised. Trocars are then inserted through the GelPOINT V-Path® allowing laparoscopic instruments to be introduced. A 20° Trendelenburg position then frees the pelvis from the bowel (Figure 1).

For the TVNH technique, the first step is the placement of the vaginal pad. The colpotomy and the transection of sacro-uterine ligaments are performed entirely endoscopically.

**Figure 2:**
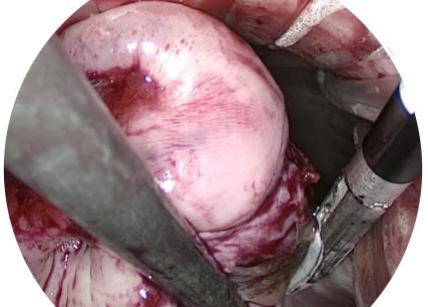
Coagulation the uterine vessels transperitoneally under visual control.

The third step is the hysterectomy (Figure 2). The surgery is performed by transvaginal laparoscopy with visual control. Firstly, one fenestrated forceps and one fenestrated bipolar forceps are used. The ureters are identified, bowel loops are mobilised out of the pelvis, and the entire abdominal cavity is inspected. The fenestrated forceps grasps the cervix and pushed it cranially and contralaterally to the side to be operated. That allows the fenestrated bipolar forceps to grasp the caudal part of the broad ligament and coagulate the uterine vessels transperitoneally. Secondly, the Voyant Maryland Fusion device® (advance bipolar forceps) is used instead of the bipolar forceps. The broad ligament is sealed and cut caudally to cranially with the advanced bipolar forceps while the pressure is maintained contralaterally by the fenestrated forceps. It is important to perform an accurate coagulation of the vessels at the beginning of the procedure because if the coagulation is not perfectly done, the uterine artery can retract behind the ring of the V-NOTES port. To access the bleeding vessels that are retracted on the sidewall of the pelvis, the ring needs to be removed to perform the haemostasis. For the rest of the uterus dissection, V-NOTES allows a good visualisation and when needed haemostasis can be performed under visual control. The surgery continues until the level of the round ligament with or without removing the ovary. The round ligament is initially left in place to avoid tilting of the uterus when working on the other side. The Fallopian tubes are removed in all women and the ovaries are removed when indicated.

The fourth step is the removal of the specimen and the V-NOTES port. Afterwards the vaginal cuff is closed by a running suture.

**Figure 3:**
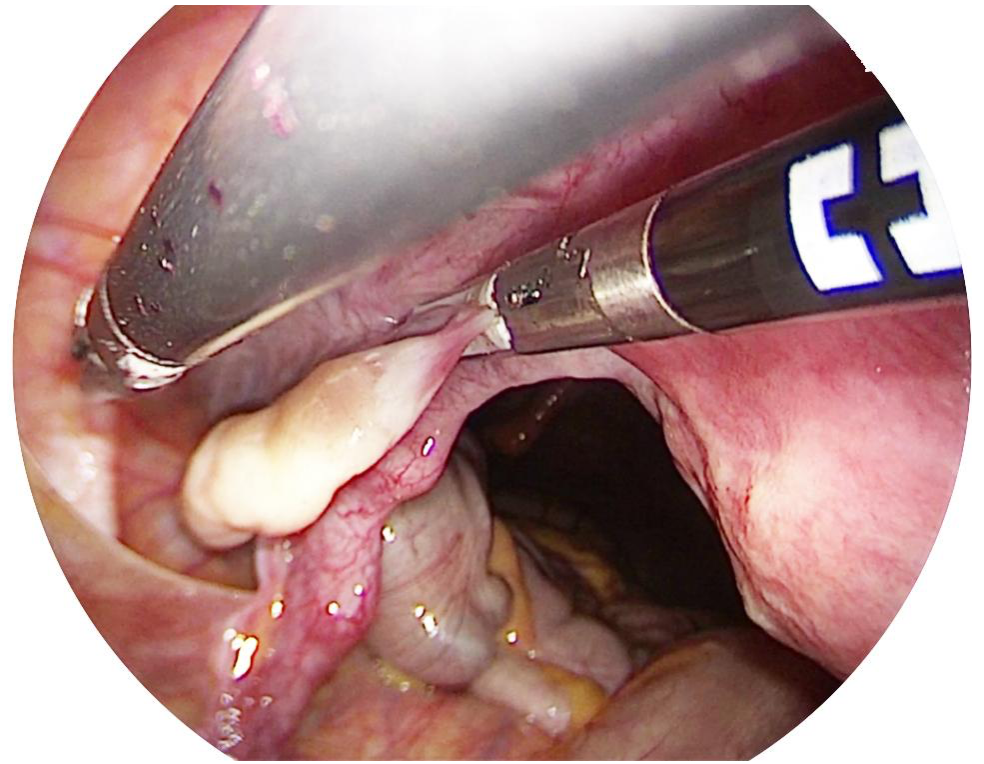
Coagulation section of the uteroovarian ligament under visual control.

To perform the adnexal surgeries by V-NOTES, the abdominal cavity is reached by a posterior colpotomy (Figure 3). In this case, the V-NOTES port (The Alexis Retractor ® retractor and GelPOINT V-Path®) is smaller but placed through the vagina in the same way as for the hysterectomy. Salpingectomy or bilateral salpingo-oophorectomy are performed using advanced bipolar endoscopic instruments.

The data was collected retrospectively. All the surgeries performed by V-VOTES were included in this study.

## Results

The demographic and clinical characteristics of the 32 patients are shown in Table I. The results are expressed as the mean +/- standard deviation with confidence interval.

**Table I:** Demographic and clinical characteristics.

Patient	Surgery	Age (years)	BMI	Vaginal delivery	History of caesarean
1	VANH	46	20,2	5	no
2	VANH	39	24,6	4	no
3	VANH	41	19,4	2	no
4	VANH	44	21,4	1	No
5	VANH	45	25,7	0	No
6	VANH	44	22	2	No
7	VANH	41	33,5	6	No
8	VANH	44	22,2	0	No
9	VANH	48	28,4	3	No
10	VANH	40	26,2	2	No
11	VANH	43	21,5	4	No
12	VANH	44	20,8	1	No
13	VANH	43	30,1	1	1
14	VANH	50	29	1	No
15	VANH	36	22,3	2	No
16	VANH	49	24,4	2	no
17	VANH	46	25,9	4	no
18	VANH	44	30,7	2	no
19	VANH	43	28,3	1	no
20	VANH	45	26,9	2	no
21	VANH	49	30	2	no
22	VANH	47	35	2	no
23	VANH	52	37	3	no
24	VANH	51	23,5	1	no
25	VANH	40	32,5	4	no
26	VANH	33	26,8	5	no
27	BS	36	35,6	3	no
28	BS	43	35,9	4	1
29	BSO	40	25,8	2	no
30	BS	39	40,2	1	no
31	BS	48	24,9	1	no
32	BS	39	22,7	3	no
Results		43,5 +/- 4,5 (33,0-52,0)	27,3 +/- 5,5 (19,4-40,2)	2,4 +/- 1,6 (0,0-6,0)	

bilateral salpingectomy (BS) - bilateral salpingo-oophorectomy (BSO)

Twenty-six patients underwent VANH, and 6 patients had adnexal surgery such as bilateral salpingectomy (BS) or bilateral salpingo-oophorectomy (BSO). The perioperative data analysed are shown in Table II.

**Table II:** Perioperative results.

Patient	Conversion	Operating time (min)	Uterus weight + tubes (g)	Duration of hospital stay (days)	Per-operative complications	Post-operative complications
1	No	55	105	2	no	no
2	no	75	135	2	no	no
3	no	80	290	2	no	Cystitis D+4
4	no	70	139	2	no	no
5	no	60	162	2	no	no
6	no	60	351	2	no	no
7	no	60	98	2	no	no
8	no	75	142	2	no	no
9	no	55	370	2	no	no
10	no	75	95	2	no	no
11	no	45	144	3	no	no
12	no	65	112	2	no	no
13	no	140	172	2	bladder injury	no
14	no	90	175	2	no	no
15	no	60	194	2	no	no
16	no	55	244	2	no	no
17	no	75	228	2	bleeding 300cc	no
18	no	60	447	2	no	no
19	no	48	288	2	no	no
20	no	42	100	2	no	no
21	no	70	310	2	no	no
22	no	55	97	2	no	no
23	no	60	133	2	no	no
24	no	115	575	2	no	infection of the surgical site D+7
25	no	90	960	2	no	no
26	no	45	136	3	no	no
27	no	80	/	1	no	no
28	no	40	/	1	no	no
29	no	25	/	1	no	no
30	no	30	/	1	no	no
31	no	40	/	1	no	no
32	no	30	/	1	no	no
Results		63,3 +/- 23,9 (25,0-140,0)	238,5 +/- 190,5 (95,0-960,0)	1,9 +/- 0,3 (1,0-3,0)	

The mean age of the patients was 43.5 +/- 4.5 years. The mean BMI was 27.3 +/- 5.5 with the highest BMI at 40.2, a patient with severe obesity.

Most of the patients had history of vaginal delivery, but 2 patients were nulliparous.

All hysterectomies were performed for benign diseases. The indications were: adenomyosis, fibromas or meno-metrorrhagia resistant to conservative medical treatments.

None of these procedures required conversion to laparoscopy or laparotomy. The average duration of the intervention for VANH is 68.5 +/- 21.8 minutes and 40.8 +/- 20.1 minutes for adnexal surgeries. The average weight of the operative specimens for the VANH is 238.5 +/- 190.5 grams. The biggest uterus weighed 960g. The surgeon progressively performed hysterectomies for larger uteri, according to the evolution of his learning curve.

The length of hospitalisation for VANH is 2.1 +/- 0.3 days and one day for adnexal surgeries.

There were 2 intraoperative complications, both occurred for hysterectomies. The first was a bleeding of the uterosacral ligament, controlled after coagulation of the bleeding’s source. The second complication was a bladder injury. We started the dissection of the vesico-vaginal plane by a conventional vaginal access but realising the difficulty of this dissection, we decided to place the V-NOTES port to perform the dissection under endoscopic control. However, despite this, the bladder was injured. After the bladder lesion, we were able to find the right dissection plane of the vesico-vaginal space. We then sutured the bladder with V-NOTES. The rest of the procedure was done with V-NOTES. At the end of the procedure, we removed the V-NOTES port. Then, the urologists performed a cystoscopy and a second suture over the bladder injury by a vaginal access. A foley catheter was left in place 7 days.

Two patients required postoperative antibiotic treatment. One for a cystitis that occurred 4 days after the surgery and the other for an infection of the surgical wound (without abscess) that occurred 7 days after the surgery. Furthermore, there was no dehiscence of the vaginal scar. There were no other complications reported at the post-operative visit 4 weeks after the surgery.

The total complication rate is 12.5 % of which 6.25 % were intraoperative complications and 6.25 % were post-operative complications. It should be noted that intraoperative complications were managed by V-NOTES and had no long- term repercussions. Postoperative complications are grade 2 according to the Clavien-Dindo classification.

## Discussion

In this retrospective study we confirmed the feasibility of V-NOTES for benign gynaecological surgery. There was no conversion. All surgeries were performed according to the planned technique without conversion.

V-NOTES combines the advantages of vaginal surgery and laparoscopy. First, vaginal access gives patients an aesthetic benefit by avoiding abdominal scars. It also avoids complications related to the abdominal trocars (hernia, wall infection, abdominal pain). The transvaginal approach also offers the advantage of relying less on possible abdominal adhesions after previous surgeries. Indeed, these ones are commonly situated in the abdomen and not in the pelvis. If needed these adhesions can be easily removed using V-NOTES.

As the vaginal route is the minimal access of choice for hysterectomy, in the era of the development of minimally invasive surgery, laparoscopy was added to the vaginal route to make the best use of this access route. Studies show that transvaginal endoscopy widens the indications of vaginal surgery by exceeding its limitations and allows more patients to gain access to the vaginal surgery route. Indeed, this technique is feasible for patients nulliparous, with a large uterus, a non- prolapsed uterus, a narrow vagina, or a history of caesarean sections.

In our series, the mean BMI was 27.3 +/- 5.5 with the highest BMI at 40.2. 10 patients were obese, but we did not encounter any difficulty or complication related to weight. Kaya et al. (2022) showed that the V-NOTES technique is applicable in obese patients and shows some advantages over TLH including shorter operating duration and postoperative hospitalisation and lower pain scores.

Another major advantage of this technique is the possibility to perform adnexal surgery. V-NOTES allows the exploration of the entire abdominal cavity, an easy access to the adnexa and an accurate dissection. Adhesiolysis (Baekelandt, 2015), salpingectomy or adnexectomy can be performed simultaneously. In our series, salpingectomy was done systematically during the hysterectomy thanks to the easy access to the adnexa. We do not currently use adhesions barrier after hysterectomy. But should studies warrant it in the future, Hyalobarrier® could be used for adnexal surgeries especially if fertility needs to be preserved.

Regarding the size of the uterus, a retrospective study (Temtanakitpaisan et al., 2018) assessed the feasibility of performing V-NOTES hysterectomy in various uterine sizes ranging from less than 500 grams to more than 1000 grams. Assuming meticulous surgical technique is employed, V-NOTES helps to approach large uterus. In contrast to VH, the uterus is pushed up towards the upper part of the abdomen. This move helps to gain space in the pelvis. The largest uterus removed with V-NOTES weighed 3361 grams (Nulens et al., 2020). In our series, the largest uterus removed weighed 960 grams. The operating time was a bit longer than the mean time with a duration of 90 minutes. However, we did not have any complications.

In these cases, the extraction of the specimen often requires morcellation of the uterus. Morcellation is only performed at the end of the procedure, in contrast to VH where the morcellation usually occurs during the surgery. Most of the specimen were extracted transvaginally at the end of the surgery without the need of morcellation. Three uteri had to be morcellated in our series and it was done without placing the specimen in a bag. We morcellated the uteri with a cold blade outside the abdominal cavity at the level of the external part of the ring. To have an optimal prevention of cell dispersion, it would be best to place a bag around the uterus before morcellation, but this step can be technically difficult. There are now specially designed bags to morcellate the specimen in via V-NOTES and we have adapted our practice accordingly.

The limitation of this technique remains the exploration of the anterior part of the bladder because of the use of a rigid scope. The development and innovation of technologies could make it possible to overcome these limits.

The length of hospital stay, and recovery are shorter. A systematic review and meta-analysis compared hysterectomies performed by V-NOTES and LAVH (Baekelandt et al., 2017). Up to 163 V-NOTES hysterectomies and 297 LAVH were analysed in 2 retrospective cohort studies. This meta-analysis shows that with V-NOTES the length of hospital stay is shorter, the patients have less postoperative pain, and the postoperative recovery is faster. This allows more patients to benefit from a hysterectomy in a day-case setting. In this study, the hysterectomies were not performed in a day case setting since the specific framework for one day hospitalisation for hysterectomy had not yet been put in place in our institution. This will be the subject of our next prospective study: the establishment of V-NOTES hysterectomy in a day case setting.

Finally, this technique is more ergonomic and allows operations to be performed with the help of a single assistant.

However, there are some contraindications to perform this technique. As the way of entering the abdominal cavity is a colpotomy, situations that bring a hazardous access to the posterior fornix should be avoided. History of rectal surgery, pelvic radiotherapy, suspected recto-vaginal endometriosis, history of pelvic inﬂammatory disease, active lower tract infection, and pregnancy are contraindications for the V-NOTES technique. Therefore, a meticulous history and vaginal exam are important to avoid peri-operative complications or conversion.

This technique does not cause more complications or postoperative readmissions. In a series of 1000 V-NOTES interventions (Baekelandt and Kapurubandara, 2021) (hysterectomies, adnexectomies and salpingectomies), the total complication rate was 3.9% including 1% intraoperative complications (cystotomy and haemorrhage requiring transfusion) and 2.9% postoperative (cystitis, hematoma, nausea and vomiting, scar infection, etc.). Note that cystotomy is a specific risk to V-NOTES hysterectomy (1.2% of cystotomy in the V-NOTES hysterectomy group for 0% in the group including other V-NOTES interventions). Our cystotomy complication occurred because of a poor vaginal access and the experience of the surgeon at the beginning of his learning curve for V-NOTES. After this complication, we have moved our practice to the use of the vaginal port during the dissection of the vesico-vaginal space when the anterior access is tricky. When the V-NOTES technique is mastered, this complication is much less frequent, and the dissection of the vesico-vaginal space can be easily done by V-NOTES. Indeed, the endoscopic vision and the insufflation can help dissection and overcome the difficulties encountered in conventional vaginal surgery. However, planned first cases utilising the V-NOTES approach should be easy cases with good vaginal access and without history of caesareans. At the beginning, when the dissection of the vesico-vaginal space seems complex during anterior colpotomy, it would be prudent to convert to laparoscopy to avoid a bladder injury complication. With higher case experience and the development of technical skills, more complex cases can be considered.

Concerning our complication rate of 12.5%, these results are related to the small number of patients included in the study. It should also be considered that the surgeon is at the beginning of his learning curve and that he progressively performs surgeries with more and more difficulties.

To analyse complications during V-NOTES procedures an iNOTES Complication Registry was created by the international NOTES society. All surgeons performing NOTES interventions are invited to register their procedures to this registry. This society currently collects all the information transmitted during the NOTES procedures. These data could be used to perform studies analysing the safety and reproducibility of the technique.

Concerning the learning curve analysis of V-NOTES hysterectomy, Wang described 4 phases. For surgeons trained in vaginal and laparoscopic surgeries, 20 cases are necessary to acquire basic skills. This is the initial learning phase. Eighty additional cases are needed to consolidate the technique, this is the skills acquisition phase. In our series, the surgeon was in phase 2 for the last cases. We have seen a trend towards shorter operating times for easy hysterectomies and increasingly complex surgeries with larger uteri. Our mean operating time for hysterectomies is 68.5 +/- 21.8 (42.0-140.0). This duration is like those observed in several other published series (Housmans et al., 2020).

Since the operative technique is well standardised, the surgery is accessible to any surgeon with experience in laparoscopy and vaginal surgery. Depending on the experience of the surgeon, either the vaginal approach or the use of laparoscopic instruments could be the most challenging part. Training in a reference centre would be the key to make this surgery reproducible and safe.

Transvaginal endoscopy has not yet become a routine technique because its evolution has never been so advanced. Other more diagnostic techniques have been developed previously. Culdoscopy was pioneered by Decker in 1939. This technique allows exploration of the pelvis with an endoscope introduced through a trocar placed in the posterior vaginal pouch. It is mainly a diagnostic technique that allows the detection of adhesions, ectopic pregnancies, salpingitis and endometriosis. Culdoscopy allows the performance of minor procedures such as tubal sterilization. Subsequently, transvaginal hydro laparoscopy was developed by Gordts and modified by Watrelot in the 1990s. Access to the pelvis is also provided by a trocar in the posterior vaginal pouch but here a saline solution is used for distension. This technique allows direct visualisation of the pelvis, especially in cases of infertility. It allows the diagnosis of pelvic gynaecological pathologies and the evaluation of tubal function. An operative port in the vaginal trocar allows adhesiolysis, ablation of endometriosis and ovarian drilling to be performed. V-NOTES now allows adnexal surgeries to be performed by a vaginal access.

Concerning the cost, the V-NOTES technique uses expensive disposable surgical instruments such as the advanced bipolar forceps (Voyant Maryland Fusion device®) and the vaginal port (GelPOINT V-Path®). The V-NOTES technique can surpass the limits of VH but at the disadvantage of a higher cost. Regarding comparison to laparoscopy, the V-NOTES technique is also more expensive from a material point of view, but it could be cheaper overall by decreasing the total cost of the hospital stay. Indeed, at present in our institution, TLH and V-NOTES hysterectomies are hospitalised for 2 days. We are currently carrying out a prospective study to introduce V-NOTES hysterectomies in one-day setting now that we have validated the feasibility and the safety of this technique in our department with an overnight stay. Baekelandt et al. (2019) mentions in his randomised study comparing V-NOTES to TLH that there is no difference in the direct health-related costs incurred up to 6 weeks after the hysterectomy based on the hospital bill.

Considering the prospects of using V-NOTES in developing countries, it might be possible to introduce this technique with “a poor man’s” NOTES. Baekelandt (2015) performed 10 V-NOTES hysterectomies with an inexpensive self-constructed single port device made with a glove and conventional reusable laparoscopic instruments. The development of a reusable and sterilisable V-NOTES vaginal ports could be interesting to reduce the cost of this technique. V-NOTES could then be used in low-income countries when VH is not feasible.

With the numerous recent publications demonstrating the advantages of V-NOTES, the access to learning and the development of surgical equipment, we can only hope to see this technique develop even more.

This technique opens the door to other gynaecological surgeries such as myomectomy, sacrocolpopexy, ovarian cystectomy and gynaecological oncology. Occasionally, treatment of ectopic pregnancies (salpingectomy or salpingotomy) can be performed by V-NOTES (Baekelandt and Vercammen, 2017).

## Conclusions

Our study demonstrates that V-NOTES procedures are feasible and safe. V-NOTES widens the limits of vaginal surgery by offering more accurate vision and making adnexal surgeries feasible.

V-NOTES is a part of the continuum of innovations in minimally invasive surgery. We cannot predict whether this developing technique will be integrated into routine clinical practice, but the research being generated seems to suggest a promising future in gynaecological surgery. Indeed, thanks to its recognised advantages, more and more surgeons are interested in this technique. We do believe that good knowledge of vaginal and laparoscopic surgery is a prerequisite. Moreover, prior theoretical learning and training are certainly the best way to perform V-NOTES accurately and safely. Further prospective multicentre randomised studies are needed to strengthen the validity of this technique.
